# Impaired Chemosensitivity of Mouse Dorsal Raphe Serotonergic Neurons Overexpressing Serotonin 1A (Htr1a) Receptors

**DOI:** 10.1371/journal.pone.0045072

**Published:** 2012-09-13

**Authors:** Gilda Baccini, Boris Mlinar, Enrica Audero, Cornelius Thilo Gross, Renato Corradetti

**Affiliations:** 1 Department of Preclinical and Clinical Pharmacology “Mario Aiazzi-Mancini” University of Florence, Florence, Italy; 2 Mouse Biology Unit, European Molecular Biology Laboratory (EMBL), Monterotondo, Italy; University of Wuerzburg, Germany

## Abstract

**Background:**

Serotonergic system participates in a wide range of physiological processes and behaviors, but its role is generally considered as modulatory and noncrucial, especially concerning life-sustaining functions. We recently created a transgenic mouse line in which a functional deficit in serotonin homeostasis due to excessive serotonin autoinhibition was produced by inducing serotonin 1A receptor (Htr1a) overexpression selectively in serotonergic neurons (Htr1a raphe-overexpressing or Htr1a^RO^ mice). Htr1a^RO^ mice exhibit episodes of autonomic dysregulation, cardiovascular crises and death, resembling those of sudden infant death syndrome (SIDS) and revealing a life-supporting role of serotonergic system in autonomic control. Since midbrain serotonergic neurons are chemosensitive and are implicated in arousal we hypothesized that their chemosensitivity might be impaired in Htr1a^RO^ mice.

**Principal findings:**

Loose-seal cell-attached recordings in brainstem slices revealed that serotonergic neurons in dorsal raphe nucleus of Htr1a^RO^ mice have dramatically reduced responses to hypercapnic challenge as compared with control littermates. In control mice, application of 9% CO_2_ produced an increase in firing rate of serotonergic neurons (0.260±0.041 Hz, n = 20, *p* = 0.0001) and application of 3% CO_2_ decreased their firing rate (−0.142±0.025 Hz, n = 17, *p* = 0.0008). In contrast, in Htr1a^RO^ mice, firing rate of serotonergic neurons was not significantly changed by 9% CO_2_ (0.021±0.034 Hz, n = 16, *p* = 0.49) and by 3% CO_2_ (0.012±0.046 Hz, n = 12, *p* = 0.97).

**Conclusions:**

Our findings support the hypothesis that chemosensitivity of midbrain serotonergic neurons provides a physiological mechanism for arousal responses to life-threatening episodes of hypercapnia and that functional impairment, such as excessive autoinhibition, of midbrain serotonergic neuron responses to hypercapnia may contribute to sudden death.

## Introduction

Serotonergic system activity participates in a wide range of basic physiological processes regulating cardiovascular and respiratory autonomic responses, arousal, sleep-wake cycle, nociception, food intake and energy balance as well as in higher brain functions such as emotion and cognition. However, the role of serotonin has traditionally been considered as modulatory and nonessential for life-sustaining responses. Recent findings using transgenic mice with functional or anatomical alterations of the serotonergic system have substantially changed this view by revealing a key role of serotonergic system in the regulation of life-sustaining autonomic functions. We created Htr1a^RO^ transgenic mice by reversible overexpression of the Htr1a selectively in serotonergic neurons [1]. This mouse line has a functional deficit in the serotonergic system due to increased negative feedback of serotonin on somatodendritic Htr1a autoreceptors. Unexpectedly, Htr1a^RO^ mice showed episodes of autonomic dysregulation, life-threatening cardiovascular crises and sudden death. A similar panel of symptoms have been found in mutant mice with structural deficits in the serotonergic system. In mice lacking serotonergic neuron-restricted Pet-1 transcription factor (Pet-1^−/−^ mice), the majority of serotonergic neuron precursors fail to differentiate while the remaining ones show multiple deficit in serotonergic-specific gene expression [2]. Pet-1^−/−^ mice exhibit increased neonatal mortality and, immediately after birth, their respiratory control is susceptible to environmental conditions, such as exposure to hypoxia or anoxia, suggesting a critical developmental window, analogous to that of sudden infant death syndrome (SIDS) [3], [4]. Another mouse line, Lmx1b^f/f/p^, in which nearly all serotonergic neurons are genetically deleted, also exhibits compromised autonomic functions, shows high mortality [5] and lacks arousal response to inhalation of CO_2_ [6]. In addition, pharmacological lesion of serotonergic neurons in neonatal rat pups, which reduced serotonin content by ∼ 80%, increased their mortality in response to repeated environmental anoxia, when tested at P7-10, suggesting a physiological role of serotonergic neurons in autoresuscitation [7].

In humans, functional and/or structural serotonergic system alterations resulting in the dysregulation of life supporting-autonomic responses are suspected to participate in SIDS, the leading cause of death for infants aged 1–12 months in developed countries [8].

Several genetic studies have revealed association of SIDS with genes involved in serotonergic function, including the serotonin transporter gene, *SLC6A4* [9]–[12], but see [13], and *Htr1a* gene [14]. Post-mortem studies have also suggested that serotonergic system abnormalities might be implicated [15]–[17] and that incomplete arousal from sleep might be the actual cause of death [8], [18]. The activity of serotonergic neurons in dorsal raphe nucleus (DRN) correlates with behavioral arousal and sleep-waking states [19]–[21]. Together with the median raphe nucleus, the DRN is considered to be part of the wake-promoting ascending arousal system (see [22]). Since acute hypercapnia is a powerful stimulus for arousal from sleep in infants and adults [23], [24] and DRN serotonergic neurons are chemosensitive [25], [26] it has been proposed that midbrain serotonergic neurons initiate the arousal response to hypercapnia and that impairment in CO_2_ chemoreception due to serotonergic system dysfunction might be the primary defect in a subset of SIDS [27].

The physiological mechanism by which altered serotonin homeostasis in Htr1a^RO^ mice compromises life-sustaining functions are unknown. We hypothesized here that excessive serotonin autoinhibition in Htr1a^RO^ mice may interfere with CO_2_ chemosensitivity of serotonergic neurons. To test this hypothesis we used loose-seal cell-attached recording to examine chemosensitivity of DRN serotonergic neurons in brainstem slices from Htr1a^RO^ mice and control littermates. We particularly focused on responses to hypercapnia, which may have a crucial role in survival response to a life-threatening event in Htr1a^RO^ mice and may be related to SIDS.

## Results

Using loose-seal cell-attached voltage-clamp recordings in brain slices obtained from Htr1a^RO^ and control mice, we compared changes in the firing rate of DRN serotonergic neurons in response to changes in P_CO2_ that reproduce *in vitro* the effects of hypercapnia (9% CO_2_) and hyperventilation (3% CO_2_). The present report is based on recordings from 31 neurons from 13 Htr1a^RO^ mice and 64 recordings from 31 control littermates.

### Intrinsic Chemosensitive Responses of DRN Serotonergic Neurons are Markedly Decreased in Htr1a^RO^ Mice

**Figure 1:**
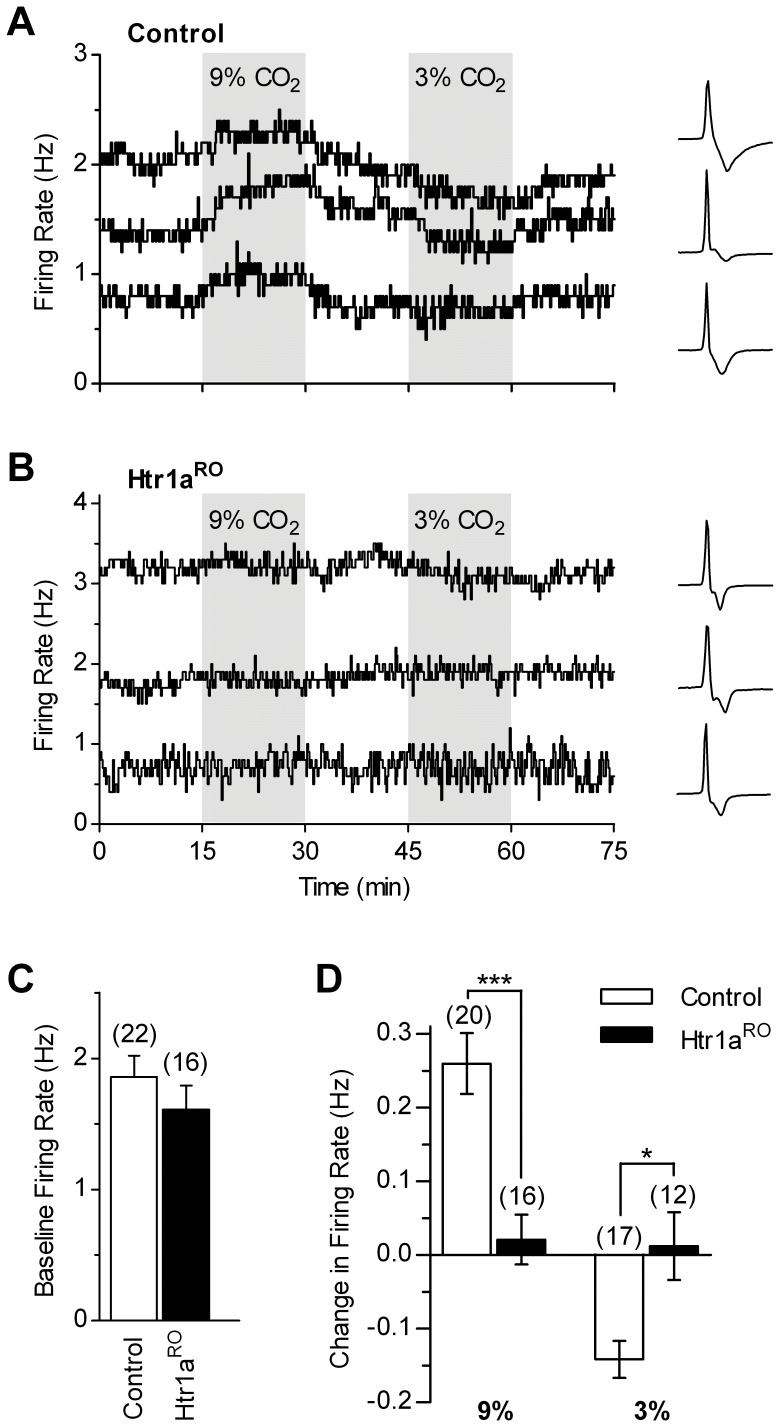
Intrinsic chemosensitive responses of serotonergic DRN neurons are greatly decreased in Htr1a^RO^ mice. *A, B,* Representative loose-seal cell-attached voltage-clamp recordings performed in the presence of synaptic blockers (see results) showing time-courses of serotonergic neuron firing in response to bath application of 9% and 3% CO_2_ in slices from control (*A*) and Htr1a^RO^ (*B*) mice. Each panel reports three time-courses from different neurons. In *B,* one neuron with basal firing rate higher than the average of Htr1a^RO^ group is shown to illustrate that the lack of responses to CO_2_ changes did not depend on basal firing rate of the recorded neuron (see results). Lines show firing rate calculated over 10 s bins. Traces illustrate recorded action currents for each experiment. *C,* Bar graph of baseline firing rate in the two groups. *D*, Summary bar graph comparing the effects of 9% and 3% CO_2_ in control and Htr1a^RO^ mice. * *p*<0.05; *** *p*<0.001 (Mann-Whitney test). Number of recorded neurons is indicated in parentheses.

To determine intrinsic chemosensitivity of serotonergic neurons in Htr1a^RO^ and control mice we measured the responses to 9% and 3% CO_2_ using artificial cerebrospinal fluid (ACSF) supplemented with a mixture of drugs containing: 10 µM phenylephrine to facilitate firing; 10 µM 2,3-dioxo-6-nitro-1,2,3,4-tetrahydrobenzo[f]quinoxaline-7-sulphonamide (NBQX) and 20 µM d-(-)-2-amino-5-phosphonopentanoic acid (d-APV) to block excitatory synaptic transmission; and 10 µM 6-imino-3-(4-methoxyphenyl)-1(6H)-pyridazinebutanoic acid (SR-95531), 2 µM 3-*N*[1-(S)-(3,4-dichlorophenyl)ethyl]amino-2-(S)-hydroxypropyl-*P*-benzyl-phosphinic acid (CGP-55845A) and 10 µM strychnine to block inhibitory synaptic transmission (Figure 1). In control mice, application of 9% CO_2_ produced an increase in firing rate of serotonergic neurons (0.260±0.041 Hz, n = 20, *p* = 0.0001) and application of 3% CO_2_ decreased their firing rate (−0.142±0.025 Hz, n = 17, *p* = 0.0008). In contrast, in Htr1a^RO^ mice, firing rate of serotonergic neurons was not significantly changed by 9% CO_2_ (0.021±0.034 Hz, n = 16, *p* = 0.49) and by 3% CO_2_ (0.012±0.046 Hz, n = 12, *p* = 0.97). The baseline firing rate of serotonergic neurons in normocapnic conditions was similar in control and Htr1a^RO^ mice (1.856±0.165 Hz, n = 22 and 1.610±0.179 Hz, n = 16, respectively, *p* = 0.45; Figure 1*C*). The responses of individual neurons to CO_2_ were not correlated with their baseline firing rate both in control (9% CO_2_, *p* = 0.11; 3% CO_2_, *p* = 0.11) and Htr1a^RO^ (9% CO_2_, *p* = 0.35; 3% CO_2_, *p* = 0.31) mice. As shown in Figure 1*D*, responses to CO_2_ in DRN serotonergic neurons from Htr1a^RO^ mice, when compared with that of control littermates, were significantly different for both 9% CO_2_ (*p* = 0.0003) and 3% CO_2_ (*p* = 0.0123) indicating marked impairment in intrinsic chemosensitivity of DRN serotonergic neurons in Htr1a^RO^ mice.

### Noradrenergic Drive is Required for Response to 9% CO_2_ in Control Mice

**Figure 2:**
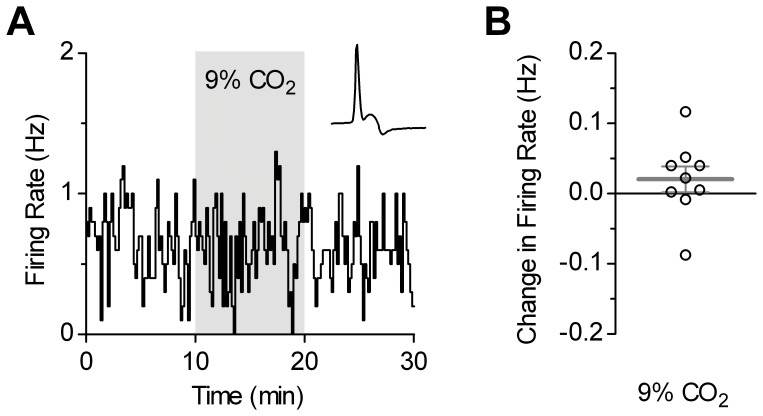
In the absence of α_1_-adrenoceptor stimulation, 9% CO_2_ does not change firing rate of spontaneously active serotonergic neurons in control mice. *A,* Time-course of a representative experiment. Phenylephrine was omitted from ACSF containing synaptic blockers. Inset shows the recorded action current. *B,* Distribution of responses to 9% CO_2_ for all recorded neurons.

It has been proposed that midbrain serotonergic neurons initiate arousal from sleep in response to hypercapnia [26]. Since facilitatory action of noradrenergic input on serotonergic neuron activity [28] is absent during sleep [29], we next investigated the response to hypercapnic challenge using ACSF supplemented with synaptic blockers, but devoid of α_1_-adrenoceptor agonist, phenylephrine. Under these conditions, most of recorded serotonergic neurons in slices from control mice were silent. As shown in Figure 2, in nine spontaneously active neurons, which had baseline firing rate of 0.460±0.150 Hz (range: 0.005–1.150 Hz), application of 9% CO_2_ did not alter their firing rate (0.020±0.018 Hz, *p* = 0.20). In additional six silent neurons, in which firing was transiently evoked by short (1–2 min) application of phenylephrine, subsequent hypercapnic challenge failed to induce any firing activity. These results suggest that noradrenergic input is essential for arousal response to hypercapnia in normal mice.

### Responses of DRN Serotonergic Neurons to Hypercapnic Challenge Persist in the Absence of Synaptic Blockade

**Figure 3:**
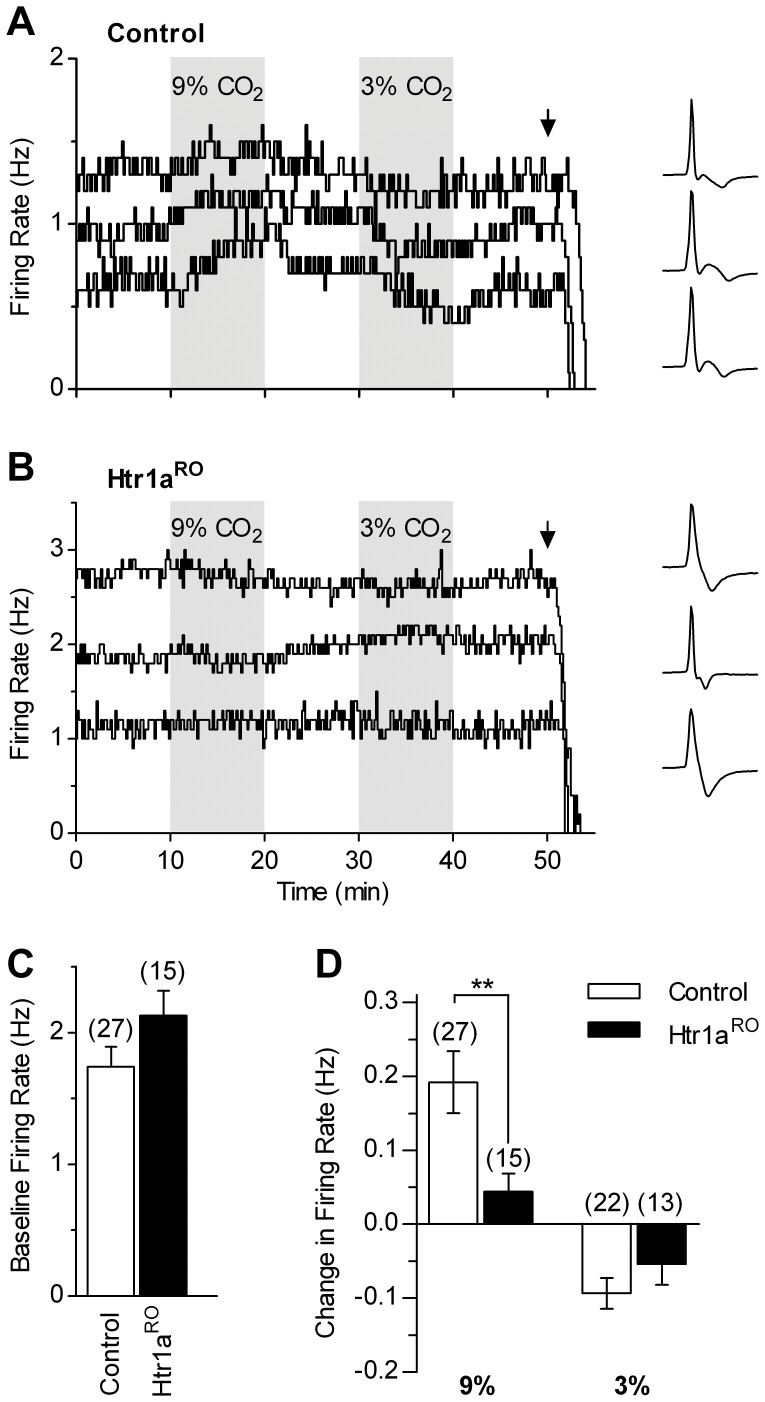
Decreased chemosensitive responses of serotonergic DRN neurons in Htr1a^RO^ mice in the absence of synaptic blockade. *A, B,* Representative recordings performed in normal phenylephrine-supplemented ACSF showing time-courses of serotonergic neuron firing in response to bath application of 9% and 3% CO_2_ in slices from control (*A*) and Htr1a^RO^ (*B*) mice. Lines show firing rate calculated over 10 s bins. Traces illustrate recorded action currents for each experiment. Arrows indicate the application of the Htr1a agonist R-8-OH-DPAT (30 nM) that silenced recorded neurons confirming that they are serotonergic. *C,* Bar graph of baseline firing rate in control and Htr1a^RO^ mice. *D,* Summary bar graph comparing the effects of 9% and 3% CO_2_ in two groups. In Htr1a^RO^ mice the response to 9% CO_2_ was significantly reduced when compared to control littermates. ** *p*<0.01 (Mann-Whitney test). Number of recorded neurons is indicated in parentheses.

We next examined serotonergic neuron chemosensitivity in conditions of preserved local network functioning, in which local mechanisms regulating serotonergic neuron activity were maintained. These experiments were done in the absence of synaptic blockade, using normal, phenylephrine-supplemented ACSF (Figure 3). In control mice, firing rate of serotonergic neurons was significantly increased by application of 9% CO_2_ (0.192±0.042 Hz, n = 27, *p<*0.0001) and significantly decreased by application of 3% CO_2_ (−0.093±0.021 Hz, n = 22, *p* = 0.0011). In contrast, firing rate of Htr1a^RO^ serotonergic neurons did not significantly change in response to 9% CO_2_ (0.044±0.025 Hz, n = 15, *p* = 0.12) and 3% CO_2_ (−0.054±0.028, n = 13, *p* = 0.13). When compared to control littermates, responses to hypercapnic challenge in Htr1a^RO^ mice were significantly decreased (*p* = 0.0097), but responses to 3% CO_2_ did not reach statistical significance (*p* = 0.24), likely due to small response in controls in this set of experiments. Taken together, the data obtained in both experimental conditions, i.e. with and without synaptic blockade, showed a marked impairment of response to hypercapnia in serotonergic neurons from Htr1a^RO^ mice (*p*<0.0001 vs. control littermates).

### Distribution of Chemosensitive Responses within the DRN

**Figure 4:**
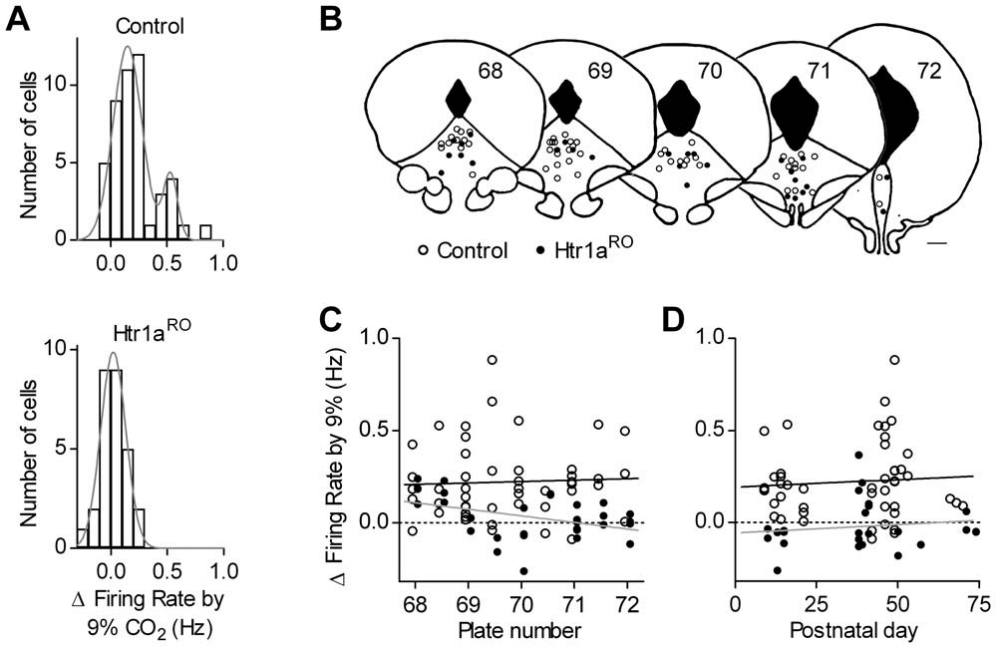
Distribution of pooled responses to hypercapnic challenge. *A*, Bar graph showing the distribution of responses in control and Htr1a^RO^ mice. Curves represent best fit of data to a double (Control, R^2^ = 0.901) and single Gaussian function (Htr1a^RO^, R^2^ = 0.952). *B,* Schematic diagram of frontal sections at various rostrocaudal levels of the DRN mouse raphe in which positions of the recorded serotonergic neurons in control (open circles) and Htr1a^RO^ mice (filled circles) are reported. Numbers correspond to plates in [39]. *C*, Individual responses to 9% CO_2_ application in slices from control (open circles) and Htr1a^RO^ mice (filled circles) plotted against the rostrocaudal position of the corresponding recorded neuron indicated by the plate number. Continuous and broken lines are best linear regressions of data in control and Htr1a^RO^, respectively. *D*, Individual responses to 9% CO_2_ application in slices from control (open circles) and Htr1a^RO^ (filled circles) plotted against the postnatal age of the mouse at time of recording. Continuous and broken lines are best linear regressions of data in control and Htr1a^RO^, respectively.

Since impairment of response to hypercapnia in Htr1a^RO^ mice may contribute to death phenotype we further examined responses to 9% CO_2_ (Figure 4). To increase population size for post-hoc analysis, data obtained in the presence of a mixture of synaptic blockers and normal ACSF were pooled. There was no significant difference in responses to hypercapnic challenge between experimental conditions (*p* = 0.15 for control, *p* = 0.74 for Htr1a^RO^). Analysis of pooled data revealed that responses in control mice did not follow a normal distribution (D’Agostino-Pearson omnibus test, *p* = 0.0049) and were well fitted with a double Gaussian function (Figure 4*A*, up). On the contrary, responses in the Htr1a^RO^ group followed a normal distribution (*p* = 0.80) and were well fitted with a single Gaussian function (Figure 4*A*, bottom). We next analyzed responses to hypercapnic challenge in respect to anatomical location of recorded neurons and to postnatal age of mice. In control mice, Spearman’s test revealed no correlation of responses (n = 47) with the rostrocaudal location of neurons (*p* = 0.47), with their lateral distance from the midline (*p* = 0.89), and with vertical distance from the aqueduct (*p* = 0.19). In Htr1a^RO^ mice, Spearman’s test revealed no correlation of responses (n = 31) with distances from the midline (*p* = 0.66) and the aqueduct (*p* = 0.25), but there was a significant correlation with the rostrocaudal location of neurons (*p* = 0.0199). An increase in firing rate in response to 9% CO_2_ was recorded in six serotonergic neurons located in the rostral margin of DRN, but not in more caudal neurons (Figure 4C). Finally, in both genotypes, responses to hypercapnic challenge did not correlate with postnatal age of mice (control, *p* = 0.63; Htr1a^RO^, *p* = 0.83, Figure 4*D*).

## Discussion

We recently reported that Htr1a^RO^ mice, in which overexpression of Htr1a autoreceptors produces excessive serotonin autoinhibition, exhibit sporadic autonomic crises that frequently progress to death [1]. The present study demonstrates that DRN serotonergic neurons in brainstem slices from Htr1a^RO^ mice have dramatically attenuated responses to hypercapnic challenge.

In control mice, firing activity of DRN serotonergic neurons was proportional to P_CO2_ both in the presence and the absence of synaptic blockers, indicating that they are intrinsically chemosensitive. The magnitude of responses was moderate, comparable to that observed by others in response to change in P_CO2_ or pH in DRN of rat [25], [26] and mice [30]. Previous evidence indicated that chemosensitivity is mild on average since it is not a property shared by all serotonergic neurons. Thus, in awake cats, only a subgroup (8 of 36) of serotonergic neurons in DRN increased their activity in response to inhalation of CO_2_ [31] and in rat brainstem slices only 16 out of 100 serotonergic neurons increased firing activity in response to hypercapnic challenge [26]. In our study, hypercapnic responses did not follow a normal distribution and were well fitted with double Gaussian function, supporting this notion. However, due to the limited sample size, the existence of a specific highly-chemoresponsive subpopulation cannot be conclusively demonstrated. If such a subpopulation exists, it is likely to be dispersed throughout DRN since analysis of hypercapnic responses in respect to anatomical location of recorded neurons revealed no evidence of a localized subgroup within the margins of dorsal and ventromedian subnuclei of DRN.

During wakefulness, serotonergic neuron activity is facilitated by noradrenergic afferents via full activation of α_1_-adrenoceptors [28] while in sleep the noradrenergic input is decreased producing disfacilitation of serotonergic neuron firing [29], [32]. In the absence of the α1-adrenoceptor agonist phenylephrine, serotonergic neurons in brainstem slices from control mice lacked the response to hypercapnic challenge, suggesting that the presence of noradrenergic drive is required for the functional response of serotonergic system to hypercapnia. This implies that during sleep, when noradrenergic input is absent, serotonergic neurons are not able to respond to hypercapnia. Beside noradrenaline, several other arousal systems, such as orexin and histamine systems, converge to excite serotonergic neurons, and we hypothesize that serotonergic system per se is insufficient to generate arousal in response to hypercapnia, and that its activity as well as chemosensitivity have to be facilitated by concurrent activity of other arousal systems.

In brainstem slices from Htr1a^RO^ mice, responses of serotonergic neurons to hypercapnic challenge were essentially abolished, except in neurons located in the most rostral margins of DRN which exhibited some residual sensitivity. Since in control mice rostral responses were similar to those in the rest of DRN there is the possibility that rostral serotonergic neurons are less sensitive to the effects of excessive autoinhibition. A more comprehensive study is needed to clarify this issue. In Htr1a^RO^ mice, death was most frequent between 25 and 80 days of life [1]. In brainstem slices from Htr1a^RO^ mice, responses of DRN serotonergic neurons to hypercapnic challenge were greatly reduced over the same time period suggesting a link between reduced chemosensitivity of serotonergic neurons and the death phenotype. It should be mentioned that susceptible period for SIDS (from birth to one year of age) appears more restricted than that for the death phenotype in Htr1a^RO^ mice. This discrepancy could derive from the fact that excessive serotonin autoinhibition in genetically engineered Htr1a^RO^ mice represents relatively persistent vulnerability factor to sudden death. Indeed, when overexpression of Htr1a was induced with the beginning at 40 or 60 days of age, significantly fewer mice died, suggesting that older mice are less vulnerable to excessive serotonin autoinhibition [1]. It is conceivable that in humans a similar vulnerability becomes crucial during susceptible period for SIDS and is later overcome by adaptive mechanisms.

The mechanism of serotonergic neuron CO2/pH sensitivity is still unknown. There is convincing evidence that chemosensitivity of serotonergic neurons derives predominantly from TASK-1 and TASK-3 channels, which are intrinsically pH chemosensitive in the physiological range [25], [30]. As in Htr1^RO^ mice, in TASK-1 and TASK-3 double knock-out (TASK^−/−^) mice there is a marked reduction in chemosensitivity of serotonergic neurons. However, different from Htr1a^RO^ mice, TASK^−/−^ mice are viable and apparently healthy, suggesting that at least in some serotonergic neurons chemosensitivity is mediated by different ion channel types or mechanisms, or alternatively altered chemosensitivity is not involved in the death phenotype of Htr1^RO^ mice. This discrepancy may also be due to methodological differences since the conclusion that TASK-1 and TASK-3 channels mediate serotonergic neuron chemosensitivity is based on whole-cell recordings, in which cytoplasm dialysis can cause loss of chemosensitivity, and in the absence of noradrenergic drive which our data suggests is critical for chemosensitivity of serotonergic neurons. Although the relationship between excessive autoinhibition and impaired chemosensitivity of serotonergic neurons in Htr1a^RO^ mice was not directly addressed in this study, it can be hypothesized that the impairment of chemosensitivity is due to a reduction in serotonergic neuron resistance via an excessive activation of GIRK channels in Htr1a^RO^ mice. Similarly, loss of chemosensitivity in the absence of the α_1_-adrenoceptor agonist phenylephrine in control mice could be caused by an analogous mechanism, since α1-adrenoceptor stimulation increases serotonergic neuron resistance *via* closure of potassium channels [33]. An alternative possibility is that serotonergic neuron chemosensitivity is mediated by a cation channel activated by α_1_-adrenoceptors.

In spite of solid evidence that at least some serotonergic neurons are chemosenstive, their importance for homeostatic control of blood pH and CO_2_ has been disputed (see [34], [35]). Recently, conditional insertional genetics was used to create a *RC::FPDi* mouse line in which the activity of serotonergic neurons can be selectively suppressed by application of a biologically inert synthetic ligand [36]. By using this approach, the authors have revealed impaired respiratory and body temperature control upon acute suppression of serotonergic neuron activity providing convincing evidence that serotonergic neurons play a key role in the central chemoreflex. Medullar serotonergic neurons innervate major respiratory centers and contribute to activity of the respiratory network, although the extent in which they mediate the ventilation responses to hypercapnia has not been clarified. Midbrain serotonergic neurons, on the other side, do not innervate major respiratory centers and are not directly involved in control of breathing. They have widespread rostral projections and are considered to be a part of wake-promoting ascending arousal system (see [22]). It has been proposed [26], that midbrain serotonergic neurons mediate non-respiratory responses to hypercapnic acidosis which are nonetheless potentially important for survival, such as induction of arousal and anxiety, and change in cerebral blood flow. Consistently, it was recently found that the arousal elicited by hypercapnic stimuli during sleep-wake transitions is abolished by genetic deletion of the serotonergic system [6]. Our findings provide additional support for a role of the serotonergic system in central chemosensitivity since they are obtained using a transgenic mice line with a structurally intact serotonergic system and only with a specific functional deficit due to increased serotonergic neuron autoinhibition. It is conceivable that the failure of DRN serotonergic neurons to respond to hypercapnia is directly or indirectly linked with the catastrophic autonomic dysregulation seen in Htr1a^RO^ mice.

The cause of terminal fatal events in SIDS victims is not yet determined. Failure to arouse from sleep during a life-threatening event could be critical for survival [37]. There is evidence indicating that infants who eventually died of SIDS exhibited incomplete arousal from sleep [18]. Considering that SIDS victims characteristically die during sleep, it can be hypothesized that impairment in responsiveness of DRN serotonergic neurons to hypercapnia contribute to incomplete arousal from sleep and loss of the resuscitation reflexes. Further investigation *in vivo* is needed to establish whether the loss of response to hypercapnia in midbrain serotonergic neurons is causally implicated in altered arousal from sleep and death in Htr1a^RO^ mice.

## Materials and Methods

All animal manipulations were performed according to the European Community guidelines for animal care (Directive 86/609/EEC) and approved by the Committee on the Ethics of Animal Experiments of the University of Florence. Mice of both sexes were anaesthetized with isofluorane and decapitated. The brain was rapidly removed, dissected in ice-cold gassed (95% O_2_ and 5% CO_2_) artificial cerebrospinal fluid (ACSF) containing (in mM): 124 NaCl, 2.75 KCl, 1.25 NaH_2_PO_4_, 1.3 MgCl_2_, 2 CaCl_2_, 26 NaHCO_3_, 11 D-glucose, and the brainstem sliced coronally into 250 µm thick slices with a DSK-T1000 vibratome (Dosaka). After recovery for 2–6 h at room temperature, the slices were individually transferred into the recording chamber and superfused continuously with warmed ACSF (34–35°C) at a rate of 2 ml min^−1^.

### Electrophysiology

Neurons were visualized by infrared differential interference contrast video microscopy with a Newicon C2400-07 camera (Hamamatsu) mounted to an Axioskop microscope (Zeiss). Recordings were made using an EPC-10 amplifier (HEKA). Patch pipettes were prepared from thick-walled borosilicate glass on a P-97 puller (Sutter) and had resistance of 3–6 MΩ when filled with solution containing (in mM): 125 NaCl, 10 HEPES, 2.75 KCl, 2 CaCl_2_, 1.3 MgCl_2_ (pH 7.4 with NaOH). Loose-seal cell-attached recordings (5–20 MΩ seal resistance) were acquired continuously in voltage-clamp mode. Signals were filtered at 3 kHz and digitized at 10 kHz. Pipette potential was maintained at 0 mV. Recordings were aborted if firing rate was sensitive to changes in pipette holding potential or if shape of action current changed. Data were analyzed using Clampfit 9.2 (Molecular Devices). Unless otherwise stated, extracellular saline was supplemented with 10 µM phenylephrine to facilitate firing [38]. Neurons were presumed serotonergic when displayed firing rate of less than 4 Hz and asymmetric action current with peak-to-peak interval (proportional to action potential half-height width) greater than 1 ms. At the end of the recording, the response to the Htr1a agonist R(+)-8-hydroxy-2-(di-n-propylamino)tetralin (R-8-OH-DPAT; 30 nM) was routinarily tested and neurons in which firing was not abolished (n = 2) were deemed unhealthy or non-serotonergic and excluded from analyses. One experiment was done in each slice. To test the effects of acid/base changes, normocapnic superfusing solution (5% CO_2_; pH 7.39, in the recording chamber) was replaced by hypercapnic solution containing 9% CO_2_ (pH 7.10) or hypocapnic solution containing 3% CO_2_ (pH 7.50). Solutions with different P_CO2_ were applied for 10–15 min and 90% of change in pH in the recording chamber was reached in ≈3 min. Steady-state values were calculated as average firing rate over the last 3–5 minutes of application and responses to 9 and 3% CO_2_ were calculated respective to firing rates measured in normocapnic solution immediately before and 10–15 min after application of CO_2_-modified solutions. Responses are expressed as difference in firing rate rather than as percent change to permit accurate quantification of the effect in slowly firing neurons, which would otherwise contribute disproportionately to average values in respect to faster firing neurons. There was no significant correlation between baseline firing rate and the change in firing rate produced by CO_2_-modified solutions (see results).

### Drugs

CGP-55845A was purchased from Tocris Bioscience. NBQX, d-APV and SR-95531 were from Ascent Scientific. All other substances were from Sigma-Aldrich.

### Statistical Analysis

Data are presented as mean and SEMs. Statistical analysis was conducted using Prism 5 (GraphPad). For assessment of significance two-tailed non-parametric tests were used: Wilcoxon signed rank test for significance of response in single groups, Mann-Whitney test for comparison between groups, and Spearman’s test for correlation between parameters. A *p*<0.05 was considered significant.

## References

[1] Audero E, Coppi E, Mlinar B, Rossetti T, Caprioli A, et al (2008) Sporadic autonomic dysregulation and death associated with excessive serotonin autoinhibition. Science 321: 130–133. 18599790 10.1126/science.1157871

[2] Hendricks TJ, Fyodorov DV, Wegman LJ, Lelutiu NB, Pehek EA (2003) Pet-1 ETS gene plays a critical role in 5-HT neuron development and is required for normal anxiety-like and aggressive behavior. Neuron 37: 233–247. 12546819 10.1016/s0896-6273(02)01167-4

[3] Erickson JT, Shafer G, Rossetti MD, Wilson CG, Deneris ES (2007) Arrest of 5HT neuron differentiation delays respiratory maturation and impairs neonatal homeostatic responses to environmental challenges. Respir Physiol Neurobiol 159: 85–101. 17656160 10.1016/j.resp.2007.06.002 PMC2593840

[4] Cummings KJ, Commons KG, Hewitt JC, Daubenspeck JA, Li A, et al (2011) Failed heart rate recovery at a critical age in 5-HT-deficient mice exposed to episodic anoxia: implications for SIDS. J Appl Physiol 111: 825–833. 21680874 10.1152/japplphysiol.00336.2011 PMC3174796

[5] Hodges MR, Wehner M, Aungst J, Smith JC, Richerson GB (2009) Transgenic mice lacking serotonin neurons have severe apnea and high mortality during development. J Neurosci 29: 10341–10349. 19692608 10.1523/JNEUROSCI.1963-09.2009 PMC2755228

[6] Buchanan GF, Richerson GB (2010) Central serotonin neurons are required for arousal to CO_2_ . Proc Natl Acad of Sci USA 107: 16354–16359. 20805497 10.1073/pnas.1004587107 PMC2941296

[7] Cummings KJ, Hewitt JC, Li A, Daubenspeck JA, Nattie EE (2011) Postnatal loss of brainstem serotonin neurones compromises the ability of neonatal rats to survive episodic severe hypoxia. J Physiol 589: 5247–5256. 21911619 10.1113/jphysiol.2011.214445 PMC3225677

[8] Moon RY, Horne RS, Hauck FR (2007) Sudden infant death syndrome. The Lancet 370: 1578–1587. 10.1016/S0140-6736(07)61662-6 17980736

[9] Narita N, Narita M, Takashima S, Nakayama M, Nagai T, et al (2001) Serotonin Transporter Gene Variation Is a Risk Factor for Sudden Infant Death Syndrome in the Japanese Population. Pediatrics 107: 690–692. 11335745 10.1542/peds.107.4.690

[10] Weese-Mayer DE, Zhou L, Berry-Kravis EM, Maher BS, Silvestri JM, et al (2003) Association of the serotonin transporter gene with sudden infant death syndrome: A haplotype analysis. Am J Med Genet Part A 122A: 238–245. 12966525 10.1002/ajmg.a.20427

[11] Okado N, Narita M, Narita N (2002) A serotonin malfunction hypothesis by finding clear mutual relationships between several risk factors and symptoms associated with sudden infant death syndrome. Medical Hypotheses 58: 232–236. 12018976 10.1054/mehy.2001.1483

[12] Nonnis Marzano F, Maldini M, Filonzi L, Lavezzi AM, Parmigiani S, et al (2008) Genes regulating the serotonin metabolic pathway in the brain stem and their role in the etiopathogenesis of the sudden infant death syndrome. Genomics 91: 485–491. 18387780 10.1016/j.ygeno.2008.01.010

[13] Paterson DS, Rivera KD, Broadbelt KG, Trachtenberg FL, Belliveau RA, et al (2010) Lack of association of the serotonin transporter polymorphism with the sudden infant death syndrome in the San Diego dataset. Pediatr Res 68: 409–413. 20661167 10.1203/PDR.0b013e3181f2edf0 PMC3242414

[14] Morley ME, Rand CM, Berry-Kravis EM, Zhou L, Fan W, et al (2008) Genetic variation in the HTR1A gene and sudden infant death syndrome. Am J Med Genet Part A 146A: 930–933. 18286597 10.1002/ajmg.a.32112

[15] Paterson DS, Trachtenberg FL, Thompson EG, Belliveau RA, Beggs AH, et al (2006) Multiple serotonergic brainstem abnormalities in sudden infant death syndrome. JAMA 296: 2124–2132. 17077377 10.1001/jama.296.17.2124

[16] Kinney HC, Richerson GB, Dymecki SM, Darnall RA, Nattie EE (2009) The Brainstem and serotonin in the sudden infant death syndrome. Annu Rev Pathol Mech Dis 4: 517–550. 10.1146/annurev.pathol.4.110807.092322 PMC3268259 19400695

[17] Duncan JR, Paterson DS, Hoffman JM, Mokler DJ, Borenstein NS, et al (2010) Brainstem serotonergic deficiency in sudden infant death syndrome. JAMA 303: 430–437. 20124538 10.1001/jama.2010.45 PMC3242415

[18] Kato I, Franco P, Groswasser J, Scaillet S, Kelmanson I, et al (2003) Incomplete arousal processes in infants who were victims of sudden death. Am J Respir Crit Care Med 168: 1298–1303. 12917226 10.1164/rccm.200301-134OC

[19] Trulson ME, Jacobs BL (1979) Raphe unit activity in freely moving cats: correlation with level of behavioral arousal. Brain Res 163: 135–50. 218676 10.1016/0006-8993(79)90157-4

[20] Sakai K, Crochet S (2001) Differentiation of presumed serotonergic dorsal raphe neurons in relation to behavior and wake-sleep states. Neuroscience 104: 1141–1155. 11457597 10.1016/s0306-4522(01)00103-8

[21] Urbain N, Creamer K, Debonnel G (2006) Electrophysiological diversity of the dorsal raphe cells across the sleep-wake cycle of the rat. J Physiol 573: 679–695. 16613874 10.1113/jphysiol.2006.108514 PMC1779756

[22] Saper CB, Fuller PM, Pedersen NP, Lu J, Scammell TE (2010) Sleep state switching. Neuron 68: 1023–1042. 21172606 10.1016/j.neuron.2010.11.032 PMC3026325

[23] Berthon-Jones M, Sullivan CE (1984) Ventilation and arousal responses to hypercapnia in normal sleeping humans. J Appl Physiol 57: 59–67. 6432751 10.1152/jappl.1984.57.1.59

[24] Franco P, Kato I, Richardson HL, Yang JSC, Montemitro E, et al (2010) Arousal from sleep mechanisms in infants. Sleep Medicine 11: 603–614. 20630799 10.1016/j.sleep.2009.12.014

[25] Washburn CP, Sirois JE, Talley EM, Guyenet PG, Bayliss DA (2002) Serotonergic raphe neurons express TASK channel transcripts and a TASK-like pH- and halothane-sensitive K^+^ conductance. J Neurosci 22: 1256–1265. 11850453 10.1523/JNEUROSCI.22-04-01256.2002 PMC6757559

[26] Severson CA, Wang W, Pieribone VA, Dohle CI, Richerson GB (2003) Midbrain serotonergic neurons are central pH chemoreceptors. Nat Neurosci 6: 1139–1140. 14517544 10.1038/nn1130

[27] Richerson GB (2004) Serotonergic neurons as carbon dioxide sensors that maintain pH homeostasis. Nat Rev Neurosci 5: 449–461. 15152195 10.1038/nrn1409

[28] Levine ES, Jacobs BL (1992) Neurochemical afferents controlling the activity of serotonergic neurons in the dorsal raphe nucleus: microiontophoretic studies in the awake cat. J Neurosci 12: 4037–4044. 1357117 10.1523/JNEUROSCI.12-10-04037.1992 PMC6575962

[29] Takahashi K, Kayama Y, Lin JS, Sakai K (2010) Locus coeruleus neuronal activity during the sleep-waking cycle in mice. Neurosci 169: 1115–1126. 10.1016/j.neuroscience.2010.06.009 20542093

[30] Mulkey DK, Talley EM, Stornetta RL, Siegel AR, West GH, et al (2007) TASK channels determine pH sensitivity in select respiratory neurons but do not contribute to central respiratory chemosensitivity. J Neurosci 27: 14049–14058. 18094244 10.1523/JNEUROSCI.4254-07.2007 PMC6673518

[31] Veasey SC, Fornal CA, Metzler CW, Jacobs BL (1997) Single-unit responses of serotonergic dorsal raphe neurons to specific motor challenges in freely moving cats. Neuroscience 79: 161–169. 9178872 10.1016/s0306-4522(96)00673-2

[32] Sakai K, Crochet S (2000) Serotonergic dorsal raphe neurons cease firing by disfacilitation during paradoxical sleep. NeuroReport 11: 3237–3241. 11043555 10.1097/00001756-200009280-00037

[33] Aghajanian GK (1985) Modulation of a transient outward current in serotonergic neurones by alpha 1-adrenoceptors. Nature 315: 501–503. 2582271 10.1038/315501a0

[34] Corcoran AE, Hodges MR, Wu Y, Wang W, Wylie CJ, et al (2009) Medullary serotonin neurons and central CO_2_ chemoreception. Respir Physiol & Neurobio 168: 49–58. 10.1016/j.resp.2009.04.014 PMC2787387 19394450

[35] Guyenet PG, Stornetta RL, Bayliss DA (2010) Central respiratory chemoreception. J Comp Neurol 518: 3883–3906. 20737591 10.1002/cne.22435 PMC2929977

[36] Ray RS, Corcoran AE, Brust RD, Kim JC, Richerson GB, et al (2011) Impaired respiratory and body temperature control upon acute serotonergic neuron inhibition. Science 333: 637–642. 21798952 10.1126/science.1205295 PMC3729433

[37] Newman NM, Trinder JA, Phillips KA, Jordan K, Cruickshank J (1989) Arousal deficit: mechanism of the sudden infant death syndrome? Aust Paediatr J 25: 196–201. 2590113

[38] Vandermaelen CP, Aghajanian GK (1983) Electrophysiological and pharmacological characterization of serotonergic dorsal raphe neurons recorded extracellularly and intracellularly in rat brain slices. Brain Res 289: 109–119. 6140982 10.1016/0006-8993(83)90011-2

[39] Paxinos G, Franklin KBJ (2001) The mouse brain in stereotaxic coordinates. 2^nd^ ed. San Diego: Academic Press.

